# PLGA nanoparticles as a platform for vitamin D-based cancer therapy

**DOI:** 10.3762/bjnano.6.135

**Published:** 2015-06-12

**Authors:** Maria J Ramalho, Joana A Loureiro, Bárbara Gomes, Manuela F Frasco, Manuel A N Coelho, M Carmo Pereira

**Affiliations:** 1 LEPABE, Department of Chemical Engineering, Faculty of Engineering, University of Porto, Rua Dr. Roberto Frias, 4200-465 Porto, Portugal

**Keywords:** 1α,25-dihydroxyvitamin D_3_, calcitriol, cancer therapy, drug delivery, poly(lactic-*co*-glycolic acid)

## Abstract

Poly(lactic-*co*-glycolic acid) (PLGA) nanoparticles were studied as drug delivery vehicles for calcitriol, the active form of vitamin D_3_. In vitro effects of calcitriol encapsulated in PLGA nanoparticles were evaluated with respect to free calcitriol on human pancreatic cell lines, S2-013 and hTERT-HPNE, and the lung cancer cell line A549. Encapsulated calcitriol retained its biological activity, reducing the cell growth. Cytotoxicity assays demonstrated that encapsulation of calcitriol enhanced its inhibitory effect on cell growth at a concentration of 2.4 μM for the S2-013 cells (91%) and for A549 cells (70%) comparared to the free calcitriol results. At this concentration the inhibitory effect on nontumor cells (hTERT-HPNE) decreased to 65%. This study highlights the ability of PLGA nanoparticles to deliver vitamin D_3_ into cancer cells, with major effects regarding cancer cell cycle arrest and major changes in the cell morphological features.

## Introduction

Vitamin D_3_, a secosteroid hormone produced through sunlight exposure [1], can be found with different chemical structures: calciol or cholecalciferol, calcidiol and calcitriol. Cholecalciferol is inert and must be metabolized in the liver and the kidney through two hydroxylation processes in order to be converted into its active form, calcitriol [2]. Calcitriol acts like classical steroid hormones, binding to vitamin D receptor (VDR) and targeting gene expression via both genomic and nongenomic pathways [1]. Although known as an important regulator of calcium homeostasis and bone mineralization [3], several studies support that vitamin D also plays a major role in tumor pathogenesis, progression and therapy [2]. Calcitriol is also regarded as a cancer chemopreventive agent due to promising epidemiological, preclinical and clinical findings [4]. The protective role of vitamin D against cancer has been mainly attributed to its anti-inflammatory activity [5].

The antineoplastic activity of calcitriol in pancreatic and lung cancer is well established, as reported by various in vitro and in vivo studies [6–13]. Several pathways by which calcitriol may prevent, treat or stop tumor growth have been described [1–2]. However, calcitriol exhibits antitumoral activity only in supraphysiological doses associated with a high risk of hypercalcemia [14]. Also, vitamin D_3_ is sensitive to many external and environmental factors such as temperature changes, oxygen pressure, light, etc. that may affect the molecular structure and the associated functionality [15]. Studies also show that more than 75% of vitamin D intake is catabolized and excreted before being converted to its active form or before its storage. In addition to these drawbacks, the clinical use of vitamin D_3_ exhibits other concerns as its short half-life in the bloodstream [16] and first-pass effect [17].

Despite multiple the medicinal benefits of calcitriol, the discussed drawbacks continue to be highlighted as major challenges in developing formulations for clinical use. To overcome some of these limitations, we propose drug delivery systems for new calcitriol formulations. These nanosystems, namely nanoparticles (NPs), must meet several requirements such as biocompatibility, biodegradability, mechanical strength, FDA approval and low synthesis complexity. One of the most attractive candidates is poly(lactic-*co*-glycolic acid) (PLGA), which is a copolymer of poly(lactic acid) (PLA) and poly(glycolic acid) (PGA) [18–19]. We expect that vitamin D_3_ encapsulation in these polymeric NPs will increase bioavailability by preventing drug degradation before administration, increasing the half-life of vitamin D_3_ in the bloodstream, avoiding the first-pass effect and circumventing the multidrug resistance (MDR) problem [18]. Also it is well documented that PLGA NPs are efficiently internalized by targeted cells, increasing intracellular drug delivery [20], allowing a sustained and controlled drug release over time [19]. Moreover, PLGA NPs could offer selective drug delivery to tumor tissue either by passive targeting with the enhanced permeability and retention effect (EPR) [18] or by active targeting, using functionalized NPs [21]. Thus, the drug toxicity on healthy cells could be reduced, increasing NPs accumulation in the target tissues [19].

Although several studies on vitamin D_3_ encapsulation for food fortification have been conducted, very few works reported the use of nanocarriers for vitamin D_3_ delivery towards cancer treatment. Vitamin D_3_ vectorisation to guarantee specific action on malignant cells that avoids side effects such as hypercalcemia has been proposed. Nguyen et al. developed a formulation based on poly(vinyl neodecanoate-crosslinked-ethyleneglycol dimethacrylate) microspheres with a size of about 35 μm [22]. In this project, the authors used cholecalciferol as a drug model for calcitriol. They demonstrated that their cholecalciferol-loaded microspheres are biocompatible, allowed for controlled and sustained release, and increased the efficiency of the therapy [22]. A few years later, Almouazen et al. developed a formulation using PLA nanoparticles of about 200 nm [14]. This study proved that PLA nanocapsules are a suitable choice for controlled delivery of antineoplastic agents, namely the nanoencapsulated calcidiol induced a significant growth inhibition when compared to free calcidiol, and the PLA NPs enhanced the intracellular delivery of vitamin in breast cancer cells [14]. In another work, Bonor et al. [23] developed calcitriol-conjugated quantum dots to analyze calcitriol distribution and dynamics in mouse myoblast cells. The authors concluded that the designed tool is suitable for imaging drug–tumor interactions and to deliver drugs to tumors and metastasized sites [23].

The aim of the present study was to evaluate the capacity of PLGA NPs to encapsulate and to deliver calcitriol, the active form of vitamin D_3_, into human cells. PLGA NPs were prepared as nanocarriers, and for the purpose of formulating and characterizing the designed system, the inactive form of vitamin D_3_, cholecalciferol, was also used as drug model along with calcitriol. We evaluated the effect of calcitriol-loaded PLGA NPs on normal and tumor cells in terms of cell growth, cell cycle arrest and morphological changes.

## Results

### Nanoparticle physicochemical properties

PLGA NPs were prepared by a single emulsion solvent evaporation method and stabilized with Pluronic^®^F127. The obtained results for mean the diameter, polydispersity index (PDI) and zeta potential for the unloaded PLGA NPs are shown in Table 1. According to the literature, the PLGA NPs size is found to be in the range of 100 to 250 nm [20]. The prepared unloaded NPs are within the expected range, exhibiting a mean diameter of 172 ± 4 nm, and presenting a zeta potential value of −38 mV: negative, as expected, due to their carboxylic end groups (Table 1).

**Table 1:** Physicochemical features of unloaded, cholecalciferol and calcitriol-loaded PLGA NPs. The data is presented as the mean ± SD (*n* = 3).

PLGA NPs	Mean diameter (nm)	PDI	Zeta potential (mV)	EE (%)	LC (%)

Unloaded	172 ± 4	0.064 ± 0.040	−38 ± 3	–	–
Cholecalciferol-loaded	187 ± 7	0.110 ± 0.065	−29 ± 3	83 ± 2	8.3 ± 0.2
Calcitriol-loaded	186 ± 3	0.056 ± 0.025	−34 ± 4	57 ± 8	5.7 ± 0.9

**Figure 1:**
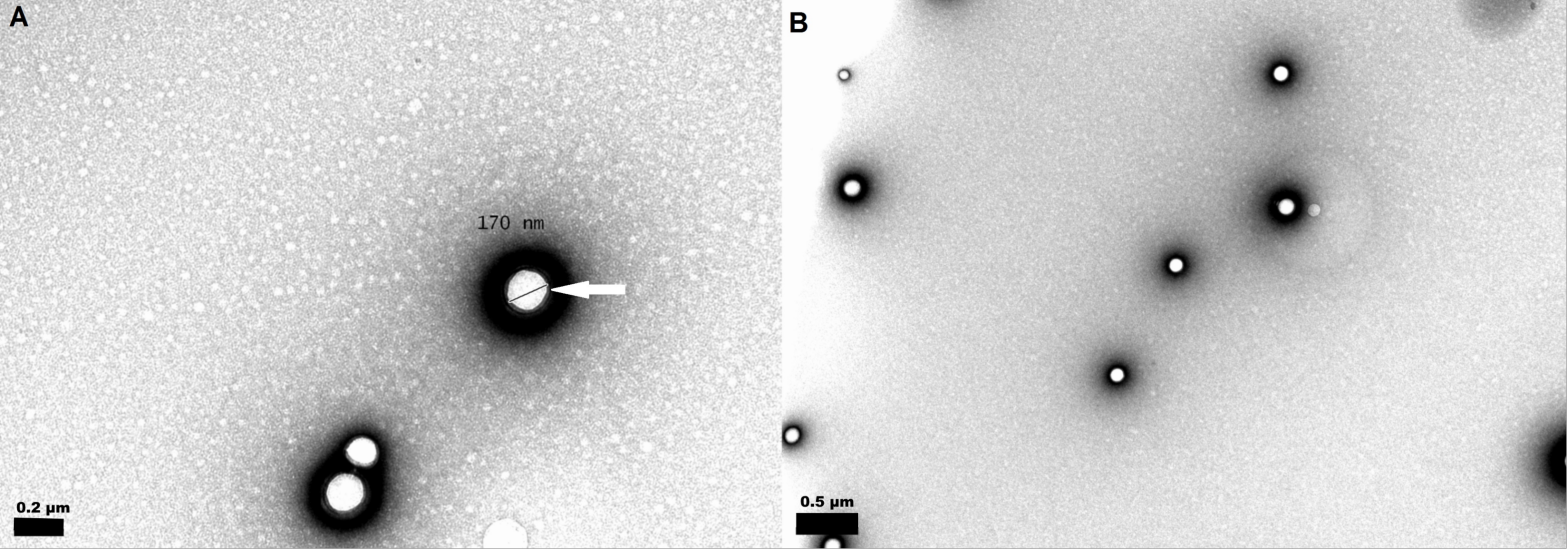
TEM images of (a) unloaded PLGA nanospheres, scale bar: 200 nm; and (b) loaded PLGA nanospheres, scale bar: 500 nm. The white arrow indicates the pluronic layer surrounding the PLGA NP.

The single emulsion solvent evaporation method allowed the encapsulation of vitamin D_3_ in the PLGA NPs. The obtained results for the mean diameter, PDI, zeta potential, encapsulation efficiency and loading capacity values for the PLGA NPs loaded with cholecalciferol and calcitriol are also shown in Table 1. The size of the vitamin-loaded NPs (187 ± 7 nm for cholecalciferol-loaded, and 186 ± 3 nm for calcitriol-loaded) increased significantly (*p* < 0.05) in comparison to unloaded PLGA NPs (172 ± 4 nm). Moreover, the mean size values were not significantly different between cholecalciferol and calcitriol-loaded NPs (*p* > 0.05). The prepared systems exhibited a narrow size distribution (PDI ≤ 0.1). TEM analysis revealed spherical- and uniform-shaped PLGA nanoparticles, as shown in Figure 1. The diameter of the nanoparticles revealed by this method varies between approximately 170 and 190 nm, which is consistent with the size measurements by DLS.

The absolute value of the zeta potential significantly decreased from 38 in unloaded PLGA NPs to 29 in cholecalciferol-loaded NPs (*p* < 0.05). The decrease to 34 mV observed for calcitriol-loaded NPs was not significant (*p* > 0.05). Moreover, zeta potential values were not significantly different between cholecalciferol and calcitriol-loaded NPs (*p* > 0.05).

The obtained results for the encapsulation efficiency (EE) for both encapsulated forms of vitamin D_3_ are presented in Table 1. The attained values significantly decrease (*p* < 0.05) from 83 ± 2% for cholecalciferol to 57 ± 8% for calcitriol. The loading capacity of PLGA NPs was also evaluated, exhibiting significant differences in the determined values (*p* < 0.05) of 8.3 ± 0.2% for cholecalciferol-loaded NPs and 5.7 ± 0.9% for the NPs loaded with calcitriol (Table 1). The yield of the PLGA NPs production process reached values of 57 ± 4% (*n* = 3).

Calcitriol-loaded PLGA nanoparticles stability studies were carried out at 4 °C over 60 days. The NPs showed a mean size of 186 ± 3 nm, which remained constant over time, exhibiting a mean *d*/*d*_0_ value of 1.0 for approximately 50 days. The *d*/*d*_0_ parameter refers to the ratio between mean diameter at each set time measurement during the 50 days and the initial NP mean diameter. After this period, the *d*/*d*_0_ ratio increased to 6.4.

The obtained results for the mean diameter, PDI and zeta potential, presented in Table 2, allowed for the assessment of aggregation or modification of the PLGA NPs properties after freeze-drying.

**Table 2:** PLGA NPs physicochemical characterization after freeze-drying experiments, with and without a cryoprotective agent. The mean size variation is expressed in terms of the ratio *d*/*d*_0_, where *d* is mean diameter after freeze-drying and *d*_0_ is the initial NP mean diameter. The data is presented as the mean ± SD (*n* = 3).

Calcitriol–PLGA NPs		Mean diameter (nm)	*d*/*d*_0_	PDI	Zeta potential (mV)

Before freeze-drying		186 ± 3	–	0.056 ± 0.025	−34 ± 4
After freeze-drying	Without cryoprotection	1591 ± 167	8.55	0.613 ± 0.370	−31 ± 1
	Sucrose 1%	193 ± 1	1.04	0.096 ± 0.028	−29 ± 3

The NP PDI values and mean diameter significantly increased from 186 ± 3 nm to 1591 ± 167 nm (*p* < 0.05) after lyophilization without the cryoprotectant agent (Table 2), showing that the freeze-drying process caused PLGA NP aggregation, resulting in high polydispersity. No significant changes (*p* > 0.05) were observed for the zeta potential values. Hence, it is possible to conclude that these PLGA NPs are not able to overcome the stress caused by the lyophilization process, leading to their destabilization and further aggregation. However, these results also demonstrated that 1% w/v sucrose preserves particle integrity after reconstitution of lyophilized PLGA nanoparticles, yielding no significant changes in the mean diameter (*p* > 0.05). However, the zeta potential values suffer a decrease in the presence of sucrose (*p* > 0.05). This could be explained by sucrose adsorption on the NPs surface.

### Calcitriol release from the PLGA nanoparticle

**Figure 2:**
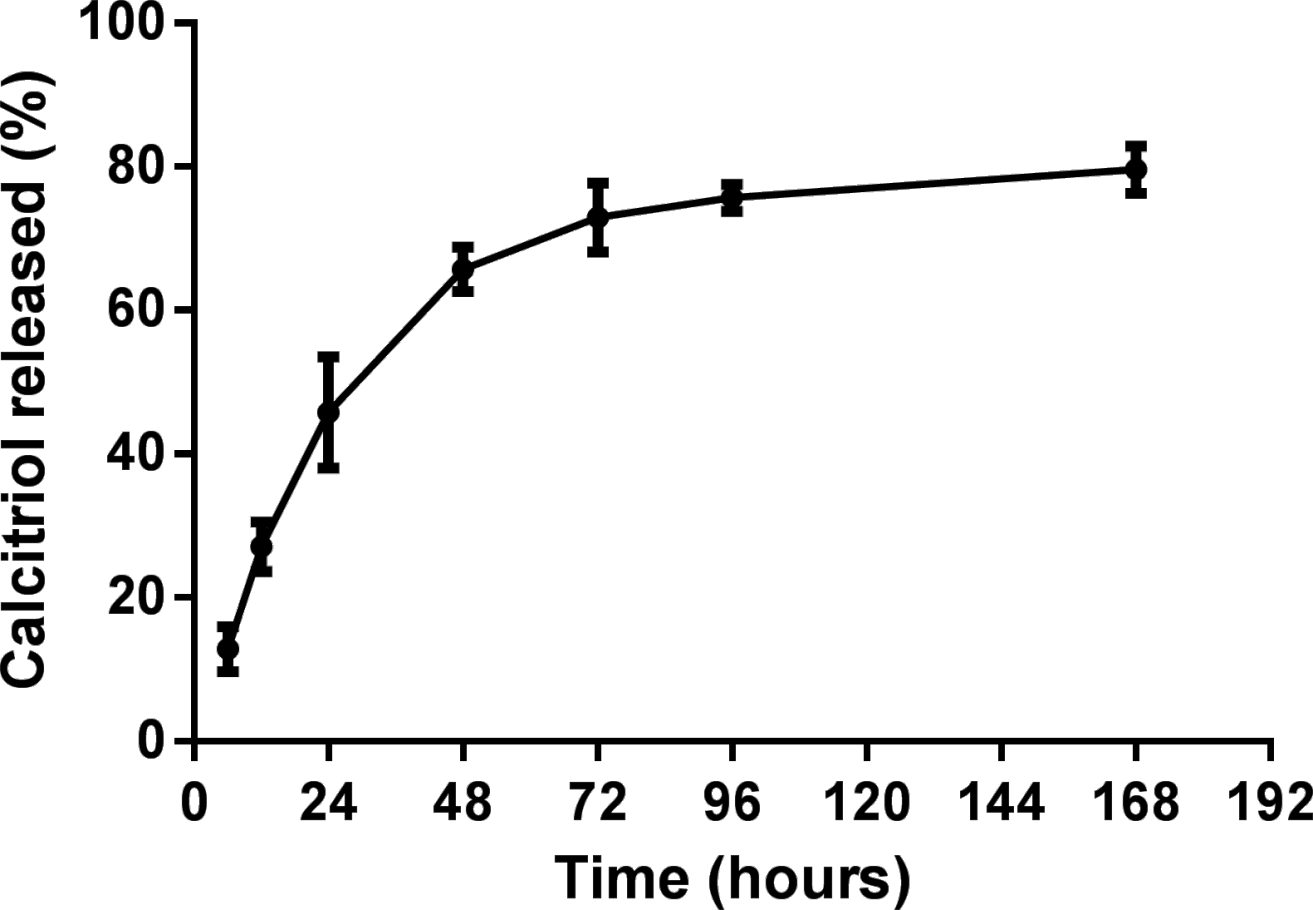
In vitro release profile of calcitriol from PLGA NPs in PBS (0.01 M, pH 7.4) at 37 °C. The data is represented as the mean ± SD (*n* = 3).

The release of calcitriol entrapped in PLGA NPs was carried out in PBS (0.01 M, pH 7.4 at 37 °C) and the results are presented in Figure 2.

The prepared PLGA NPs exhibited an initial rapid release, followed by a slower, sustained release. As Figure 2 shows, calcitriol released at 24 h was around 46%. This initial rapid release might be attributed to the release of the surface-adsorbed vitamin. The calcitriol entrapped in the polymeric matrix of the NP was released later and in a more controlled manner, reaching a quasi-plateau between 96 and 168 h. The plateau represented a release of about 4% of the encapsulated calcitriol in this period. After 168 h, the total calcitriol released was around 80%. The control sample showed that calcitriol remained stable at release conditions throughout the experiment period.

### Cellular uptake of PLGA NPs and calcitriol-induced morphological changes

**Figure 3:**
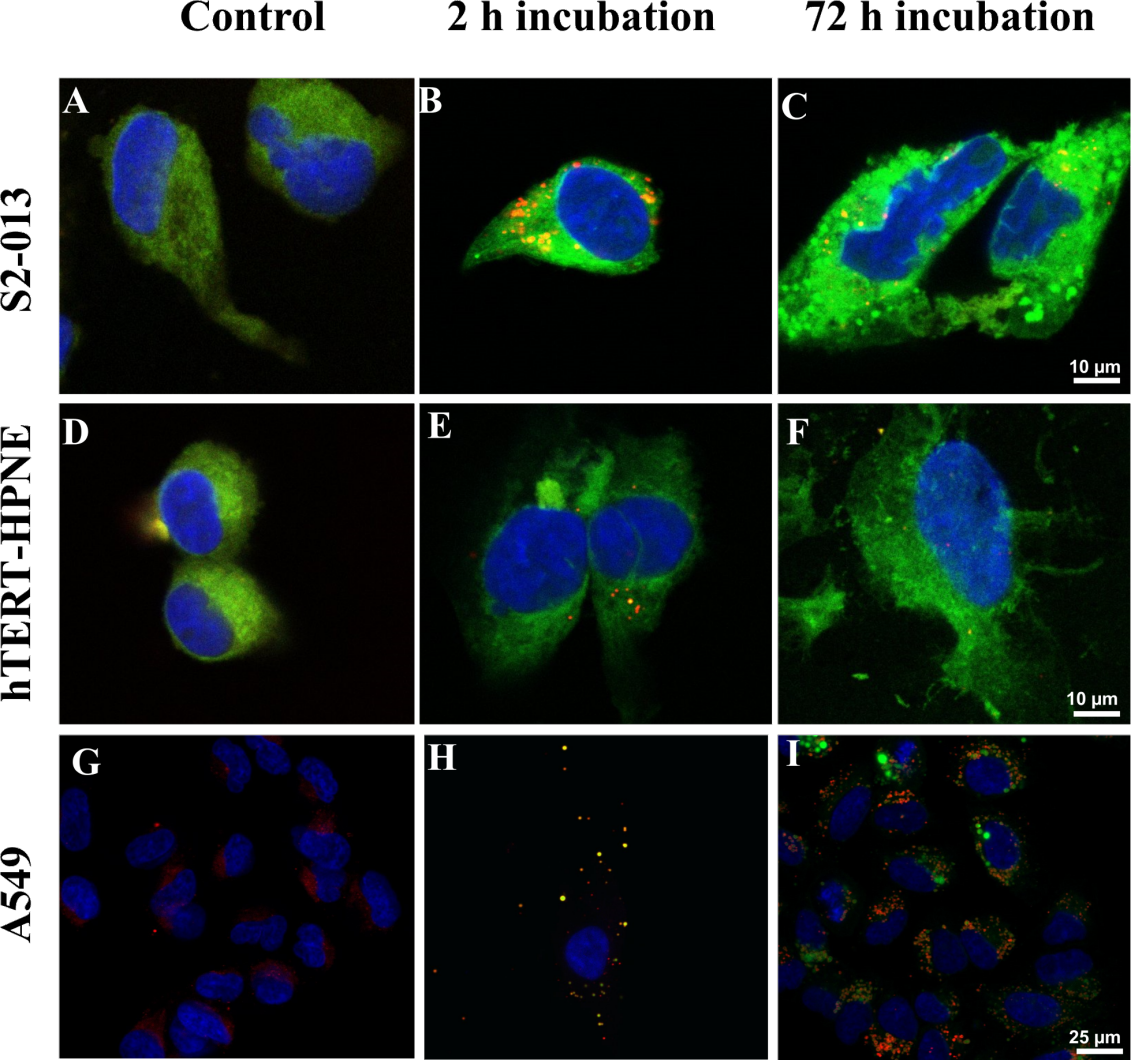
Confocal laser scanning microscopy images of human cells treated with calcitriol entrapped in C6-PLGA NPs. S2-013 cells: (A) control cells; cells after (B) 2 and (C) 72 h of incubation with C6–calcitriol–PLGA NPs. hTERT-HPNE cells: (D) control cells; cells after (E) 2 and (F) 72 h of incubation with C6–calcitriol–PLGA NPs. A549 cells: (G) control cells; cells after (H) 2 and (I) 72 h of incubation with C6–calcitriol–PLGA NPs. The blue color represents the nuclei and the yellow color represents the colocalization of PLGA NPs with the late endosomes/lysosomes. Scale bar: 10 µm for images A–F, and 25 µm from G–I.

The internalization of fluorescent C6–calcitriol–PLGA NPs by S2-013, hTERT-HPNE and A549 cells was evaluated by confocal microscopy. Counterstaining of the cell nuclei was performed with DAPI and the acidic compartments (including endosomes and lysosomes) with LysoTracker^TM^ Red. The obtained images are presented in Figure 3.

As seen in Figure 3A,D, nontreated pancreatic cells exhibit an intense green color that masks the red color of the lysotracker for lysosomes, despite not having been treated with C6. This fact is justified because both cell lines exhibited autofluorescence in the same emission spectrum as C6 and lysotracker. Lung carcinoma cells did not exhibit this intense autofluorescence, therefore allowing the visualization of the NP uptake (Figure 3G). As shown in Figure 3H, after 2 h of incubation, the nanoparticles were internalized by A549 cells. It is also possible to observe some colocalization of C6-PLGA NPs with the red-stained late endosomes or lysosomes (yellow color, in Figure 3H). Quantitative analysis with the ImageJ JACoP “colocalization finder” plug-in was used for colocalization assessment of the NPs with LysoTracker Red in the lysosomes of A549 cells [24]. The statistical method provides the Pearson coefficient (*r*), which varies between −1 (anti-colocalization) and +1 (total colocalization) and reflects the unambiguous colocalization of two fluorescent probes [25]. The Pearson correlation coefficient for A549 cells significantly decreased from 0.7 ± 0.1 for a 2 h treatment to 0.4 ± 0.1 and 0.14 ± 0.06 for 48 h and 72 h treatment, respectively (*p* < 0.05). This decrease over time suggests an endo-lysosomal escape, with most of the PLGA NPs localized in the cytoplasm after 72 h, as exhibited in Figure 3I. Due to the intense cell autofluorescence, it was not possible to determine the Pearson coefficient for both pancreatic cell lines. However it was possible to observe yellow dots in the S2-013 and hTERT-HPNE cells incubated with C6–calcitriol–PLGA NPs for 2 h (Figure 3B,E). We were able to observe this colocalization due to the higher green fluorescence intensity of C6–calcitriol–PLGA NPs. The presence of these yellow dots is reduced in cells incubated with C6–calcitriol–PLGA NPs for 72 h (Figure 3C,F), due to the endo-lysosomal escape, with most of the PLGA NPs localized in the cytoplasm. However, it is not possible to distinguish this due to autofluorescence in the pancreatic cells and extensive morphological changes. As can be seen in Figure 3C,F, pancreatic cells treated with calcitriol–PLGA NPs at 2.4 µM for 72 h displayed major changes in shape when compared to untreated growing cells (Figure 3A,D). In contrast, the control cells display rounded shapes, and the treated cells exhibited enlarged and flattened irregular shapes and multiple or enlarged nuclei, in both cell lines. The observed morphological features are consistent with senescence phenomena.

### Cell growth inhibition by calcitriol-loaded NPs

The in vitro cytotoxic effects on three different human cell lines, hTERT-HPNE, S2-013 and A549, after treatment with calcitriol entrapped into the PLGA NPs were assessed relative to free calcitriol in terms of cell growth. Treatment with 0.1% ethanol and unloaded PLGA NPs during 72 h had no significant effect on the cell growth for the used cell lines (data not showed). These results prove that PLGA nanoparticles are biocompatible.

**Figure 4:**
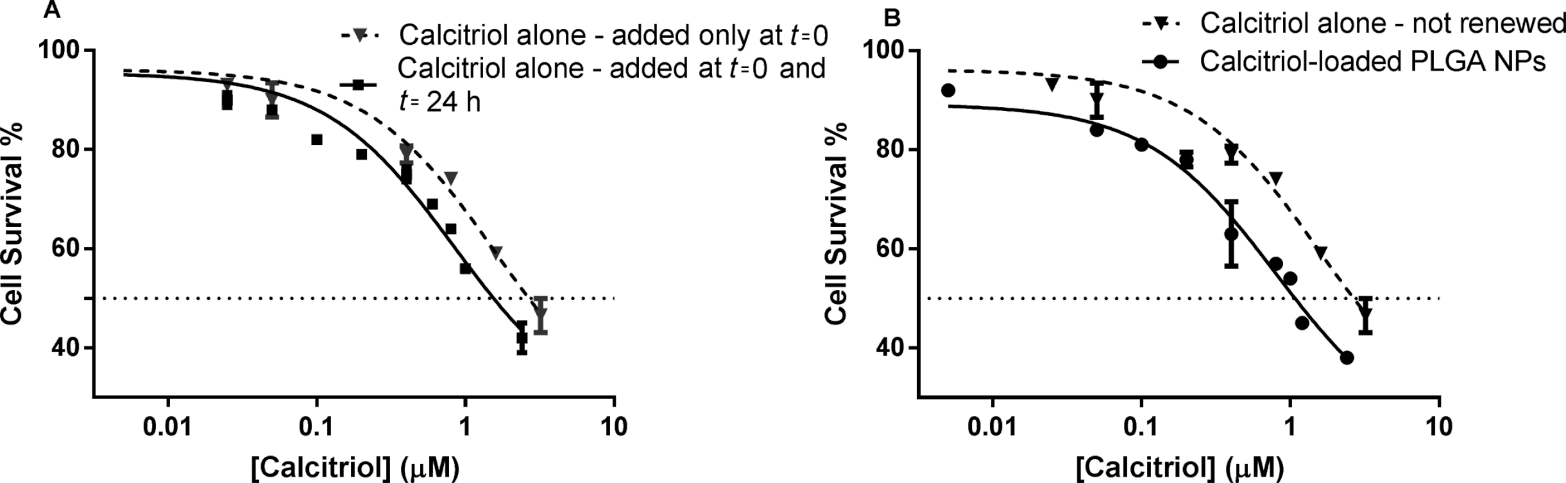
Effects of calcitriol after 48 h treatment on S2-013 cell survival, determined by SRB assay. (A) Comparison between a single-addition and daily renewal of calcitriol; (B) Comparison between calcitriol free (single-addition) and entrapped in PLGA NPs. Free calcitriol, added only at *t* = 0, is represented with triangles and a dotted line; free calcitriol, added at *t* = 0 and *t* = 24 h, is represented with squares and a solid line; and calcitriol–NPs with spheres and a solid line.

The effect of calcitriol at concentrations from 0.005 to 3.2 µM was tested with concentrations of PLGA in the range of 0.1 µg mL^−1^ to 50 µg mL^−1^. The efficacy of calcitriol-loaded NPs in comparison to free calcitriol was also evaluated. Due to its known short half-life in the cell culture medium, an assay with S2-013 cells was performed for 48 h to compare cell survival between single-addition and daily-renewed calcitriol. As shown in Figure 4A, the poor stability of calcitriol is reflected by reduced toxicity when cells are treated with a single addition for 48 h. For the same range of concentrations, single-added calcitriol at *t* = 0 shows a decreased in vitro antitumor activity when compared to daily-renewed calcitriol, resulting in significantly different (*p* < 0.05) 48 h IC_50_ values of 2.19 µM and 1.51 µM, respectively. Thus, all cytotoxicity assays with free calcitriol compared to calcitriol-loaded NPs were renewed daily.

**Figure 5:**
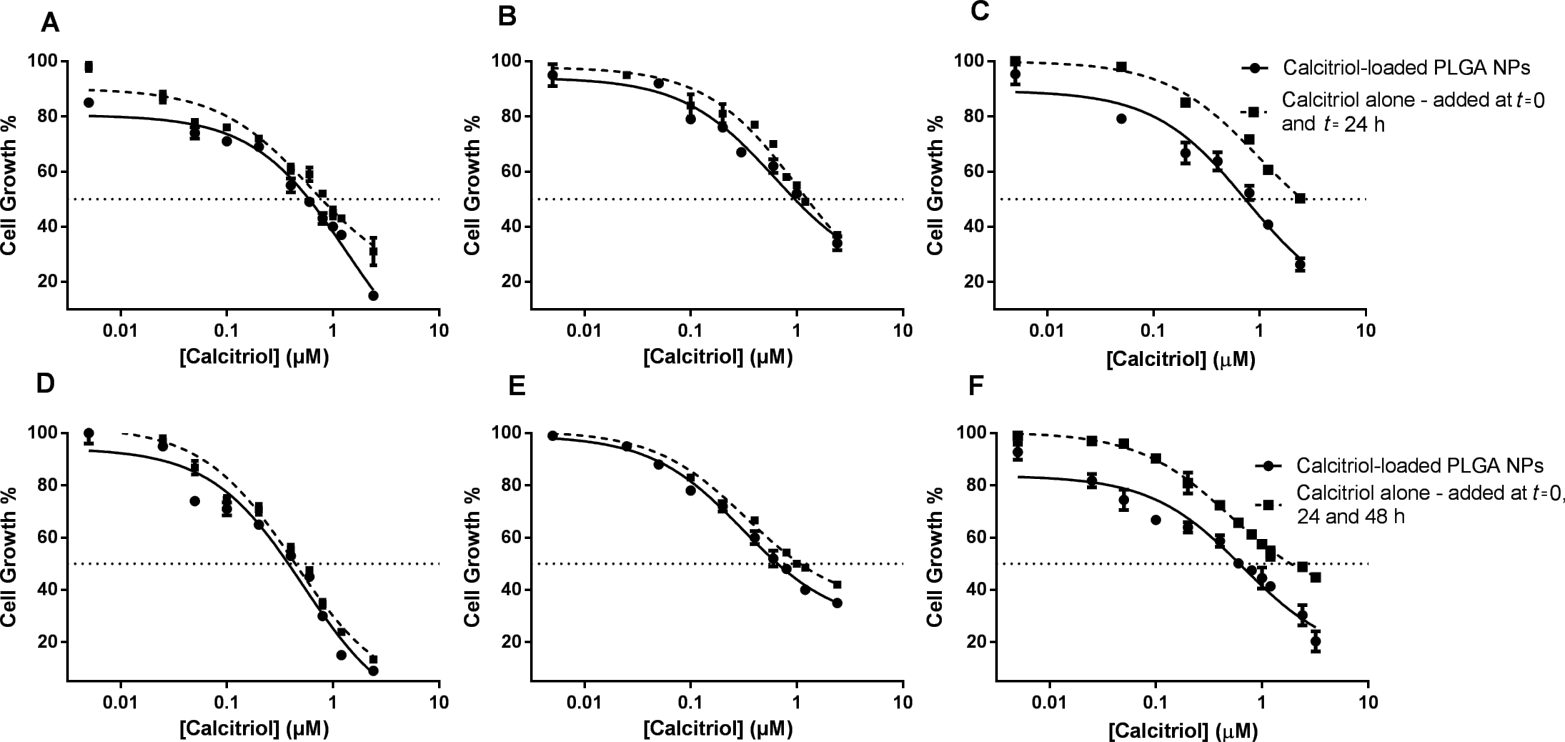
Cytotoxic effects of calcitriol free and calcitriol entrapped in PLGA NPs after 48 h (A–C) and 72 h (D–F) treatment on the cell growth of three human cell lines, (A,D) S2-013, (B,E) hTERT-HPNE and (C,F) A549, determined by a SRB assay. Free calcitriol is represented with squares and a dotted line; and calcitriol–NPs with spheres and a solid line.

Both free and encapsulated calcitriol exhibited a concentration-related decrease in cell growth and survival of the human cell lines (Figure 5, Supporting Information File 1, respectively). We observed an advantage of PLGA NPs, in that calcitriol–NPs are more efficient than free calcitriol with regards to cell growth inhibition (Figure 5). For instance, incubation for 72 h with 3.2 µM nanoencapsulated calcitriol reduced the cell growth of lung carcinoma cells to about 20% compared to 45% when free calcitriol was administered (Figure 5F). Thus, drug delivery with this polymeric system improves calcitriol antiproliferative activity, resulting in significantly (*p* < 0.05) lower GI_50_ values (Table 3). In a 48 h assay, free calcitriol inhibits the S2-013 cell growth by 50% when its concentration is 0.78 µM (renewed daily, total of 2 administrations), which is higher than equivalent 0.53 µM calcitriol of the loaded NPs (added only at *t* = 0) with the same effect (Table 3).

**Table 3:** Cytotoxic effects of calcitriol on the growth of cell lines, S2-013, hTERT-HPNE and A549, respectively. The results are expressed as GI_50_ (the concentration necessary to inhibit cell growth to 50%) at 48 and 72 h of exposure with free calcitriol and entrapped calcitriol in PLGA NPs by SRB assay.

		GI_50_ (µM)		
		S2-013	hTERT-HPNE	A549

48 h assay	Calcitriol (renewed daily)	0.78 ± 0.01	1.25 ± 0.01	2.04 ± 0.18
	Calcitriol–PLGA NPs	0.53 ± 0.02	1.12 ± 0.04	1.03 ± 0.16
72 h assay	Calcitriol (renewed daily)	0.48 ± 0.01	1.08 ± 0.01	1.90 ± 0.25
	Calcitriol–PLGA NPs	0.43 ± 0.01	0.83 ± 0.02	0.49 ± 0.10

Still, it is important to highlight that due to the short calcitriol half-life in the cell culture medium, the presented free drug concentrations were added daily, unlike entrapped calcitriol, which was loaded in the NPs only once at time *t* = 0. Thus, despite that the NPs themselves already show an advantage compared to free, daily renovated calcitriol, if the comparison was made between loaded NPs and single-addition, free calcitriol, that advantage would be much more evident as demonstrated by Figure 4B.

For the different cell types, treatment with encapsulated calcitriol for 72 h showed significantly more deleterious effects than 48 h treatment, resulting in lower GI_50_ values for the 72 h assay (*p* < 0.05) (Table 3). Also, it is quite relevant to analyze and compare the effect of free calcitriol and loaded NPs on the different cell lines. The results shown in Figure 5 demonstrated that the deleterious calcitriol effect was significantly (*p* < 0.05) higher in the pancreatic cancer cell line, S2-013. GI_50_ values for S2-013 cells are significantly lower than for hTERT-HPNE and A549 cells (Table 3) (*p* < 0.05). Although the NPs show potential for the drug’s effect on the hTERT-HPNE cell line, likewise as for the pancreatic tumor cells, hTERT-HPNE cells show more resistance to the vitamin’s toxicity, whether calcitriol is encapsulated or not. For instance, while 0.005 µM calcitriol loaded in PLGA NPs reduced the cell growth of the S2-013 cell line to about 80% after 48 h, at the same concentration, calcitriol–PLGA NPs do not show toxicity in the hTERT-HPNE cell line (Figure 5A,B). The lung carcinoma cell line exhibited the lowest sensitivity to the free calcitriol’s antiproliferative activity among the used cell lines (*p* < 0.05). However, this was not true for the encapsulated form of calcitriol. This was the cell line where the encapsulation of calcitriol in NPs proved to be more advantageous.

### Cell cycle arrest by calcitriol-loaded PLGA NPs

**Figure 6:**
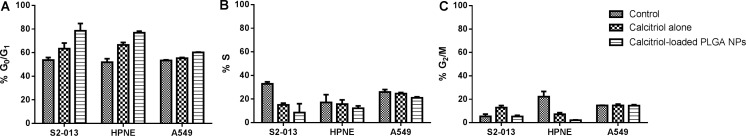
Cell cycle distribution of S2-013, hTERT-HPNE and A549 cells treated for 72 h with free calcitriol and calcitriol entrapped in PLGA NPs. The graphs show the percentage of cells in (A) G_0_/G_1_, (B) S and (Cc) G_2_/M phases. The data is represented as the mean ± SD (*n* = 3).

To assess whether the cytotoxic effects of calcitriol are due to cell cycle inhibition, cell cycle analysis by flow cytometry was performed in propidium iodide (PI)-stained S2-013, hTERT-HPNE and A549 cells after treatment with free calcitriol and calcitriol-loaded PLGA NPs at 1.2 µM for 72 h. PI counterstaining was used for DNA quantification. The differences in the DNA content between the cell population allowed the cell cycle distribution to be studied [26]. The attained results are presented in Figure 6.

As Figure 6A shows, cell cycle analysis demonstrated a significant accumulation of both pancreatic cell lines in the G_0_/G_1_ phase after exposure to calcitriol (*p* < 0.05). This accumulation was associated to a concomitant decrease in the S or/and G_2_/M phases (Figure 6B,C). Additionally, the observed changes on the cell cycle distribution between control A549 cells and A549 cells treated with free calcitriol for 72 h were not significant (*p* > 0.05). These results are in agreement with the proliferation studies, where the A549 cell line exhibited the lowest sensitivity to the calcitriol antiproliferative effect. As it is also shown in Figure 6A, encapsulation of calcitriol in PLGA NPs enhanced the calcitriol growth inhibition, inducing a significantly increased accumulation of cells in the G_0_/G_1_ phase, in the three different cell lines, compared to free calcitriol (*p* < 0.05). The cell cycle arrest in the treated cell lines was not accompanied by significant changes in the amount of sub-G_1_ cells (data not shown), relative to the control cells, indicating little apoptosis after 72 h treatment with free and entrapped calcitirol. The sub-G_1_ group represents the apoptotic cells with fractional DNA, which appear as cells with hypodiploid DNA content [27]. These data suggest that calcitriol antiproliferative effects, observed in cytotoxicity assays, could occur in consequence of cell cycle arrest.

## Discussion

The antineoplastic activity of calcitriol, the active form of vitamin D_3_, has been well documented both in vitro and in vivo. Despite calcitriol clinical application, it exhibits several limitations. This work addresses the ability of PLGA NPs to overcome some of the described drawbacks. PLGA nanoparticles were formulated as promising delivery systems to improve the therapeutic potential of calcitriol. The PLGA NPs were prepared by a single emulsion solvent evaporation method and stabilized with Pluronic^®^F127. The prepared NPs exhibited mean diameters smaller than 200 nm and negative zeta potential. The observed increase in the mean size with the encapsulation of both forms of vitamin, as compared with unloaded NPs, was anticipated. This effect was reported in studies arguing that the drug causes an expansion of the polymeric matrix, increasing the particle size [28]. The decrease in zeta potential values with the encapsulation of both forms of vitamin could be attributed to vitamin adsorption on the PLGA NPs surface. As already reported, the drug adsorbed on the PLGA NP surface exerts a masking effect of the superficial carboxylic groups, reducing the effective NP charge [28]. The NP stability is a result of electrostatic forces due to the PLGA carboxylate groups at the NP surface, and the surfactant behavior that also plays a crucial role in maintaining nanosuspension stabilization. During particle formation, the Pluronic^®^F127 is adsorbed onto the NP surface, providing steric and thermodynamic stabilization (Figure 1, white arrow) [29].

Both forms of vitamin D_3_ were used for the formulation of the nanocarrier system and variations in the encapsulation efficiency and loading capacity were noticed. The observed differences may be based on their chemical structure, since calcitriol has two extra hydroxy groups and is less hydrophobic than cholecalciferol. Thus, its partition into the aqueous phase may occur during the NP preparation, resulting in lower EE and LC values [14]. The EE and LC values achieved are in accordance with our experience with other hydrophobic drugs [30] and other results reported in literature [20]. The prepared nanoparticles remained stable under storage conditions for several weeks. The nanoparticle emulsions were successfully lyophilized by the addition of sucrose to increase the shelf-life time. The choice of sucrose as the cryoprotective agent was justified by the previous work of Holzer et al., where it was proven that this is a well-suited cryoprotectant [31].

PLGA NPs tend to exhibit a biphasic release pattern, characterized by an initial rapid release, followed by a slower sustained release [19]. As expected, the NPs exhibited a rapid release in the first 24 h due to the release of calcitriol adsorbed onto the NP surface. The sustained release over the next 168 h could be attributed to the diffusion of vitamin from the NP core into the release medium. In aqueous medium, PLGA suffers biodegradation by hydrolytic cleavage of its ester linkages into monomers. During hydrolysis, acidic degradation products accumulate inside the PLGA NPs and are responsible for reaction autocatalysis. The hydrolytic breakdown also causes the formation of pores, allowing the release of oligomers and monomers, resulting in bulk erosion [32]. As the NP degradation is slow, the release between 48 and 168 h may depend mainly on vitamin diffusion through the polymeric matrix and matrix erosion [19,33].

Human cell lines, S2-013, hTERT-HPNE and A549, were selected to evaluate the antiproliferative potential of calcitriol-loaded PLGA NPs. A549 lung carcinoma line proved to be the least sensitive line to the free calcitriol activity. Pelczynska et al. previously reported that A549 is a VDR-negative cell line, only exhibiting VDR expression after incubation with calcitriol, which explains the low sensitivity to the drug [11]. To this date, no work regarding calcitriol activity on the pancreatic cell lines, S2-013 and hTERT-HPNE, was reported. The in vitro proliferation assay showed that the encapsulation of calcitriol enhanced its antiproliferative activity. The efficient cell internalization by an endocytosis mechanism of PLGA NPs and their rapid endo-lysosomal escape observed in this study could explain the benefits of the drug encapsulation in the NPs. Tahara et al. showed that PLGA NPs are efficiently internalized by A549 cells by an endocytosis mechanism, partially mediated by a clathrin [34], which can explain the NP-enhanced calcitriol activity reported in this work. Therefore, this mechanism of NP internalization avoids calcitriol transport out of cells mediated by P-glycoprotein involved in the MDR problem. It was previously established that after internalization, PLGA NPs suffer a charge change triggered by the acidic medium of late endosome/lysosome. This leads to the destabilization of the endo-lysosomal membrane, allowing the escape of NPs into the cytoplasm [18]. Also, the prepared nanoparticles were stabilized with the Pluronic^®^F127, known for its ability to overcome MDR by direct inhibition of P-glycoprotein [35]. The obtained results suggest that these PLGA NPs are able to work as cytoplasmic delivery vehicles. Also, because calcitriol has a short half-life, its entrapment in PLGA NPs allows vitamin protection, sustained and controlled delivery, thus avoiding drug degradation and inactivation. The sustained and controlled release of the prepared PLGA NPs explains the increased inhibition of cell growth in the 72 h assay, as compared with the 48 h assay. We conclude that a longstanding treatment presents more pronounced, deleterious effects since these NPs are able to maintain drug concentrations.

Furthermore, flow cytometry analysis demonstrated that encapsulation of calcitriol in PLGA NPs enhanced the growth inhibition of the human cells by inhibiting the cell cycle progression at the G_1_–S transition, as previously reported [7,12,36–37]. As confocal imaging studies demonstrated, this cell cycle arrest was associated with major changes in the morphological features of the calcitriol-loaded PLGA-NP-treated pancreatic cells, consistent with the senescence phenomena. Senescent cells are described as cells permanently arrested in the cell cycle [38], and it was already reported that calcitriol can trigger cell senescense [39].

## Conclusion

A PLGA NP system was developed for calcitriol delivery. The prepared system is stable under storage conditions for several weeks and was lyophilized to increase its shelf life. The NPs exhibited a rapid release in the first 24 h followed by sustained release over the following days, after which a diffusion equilibrium between the NPs and release medium occurred. The in vitro cytotoxic studies proved that unloaded PLGA NPs are biocompatible and revealed the toxicity effect of calcitriol against human pancreatic and lung cells. Due to the short calcitriol half-life in the cell culture medium, daily renewal was necessary to maintain its concentration. This results in an increase in the frequency of administration, and consequently, in the increased amount of drug in comparison to calcitriol encapsulated in the PLGA nanoparticles. As a result, the obtained data prove that PLGA NPs enhance calcitriol antineoplastic activity, allowing reduced administration frequency, as well as lower drug dosage, and thus increased drug bioavailability. This work also demonstrated that encapsulation in a nanovehicle enhanced the growth inhibition effect of calcitriol in the treated human cell lines by inducing cell cycle arrest in the G_1_–S phase. This antiproliferative effect was associated with major morphological changes in the treated cells. The obtained results suggest that the lack of growth of the human cells lines upon treatment with free and entrapped calcitriol is a result of a drug-induced senescence. Thus, we can conclude that nanoencapsulation in PLGA NPs may offer a new and potentially effective administration strategy of calcitriol that overcomes the actual limitations such as its low bioavailability.

## Experimental

### Chemicals

PLGA Resomer^®^ RG503H (50:50; *M*_w_ 24,000–38,000), ethyl acetate, Pluronic^®^F127, coumarin-6 (C6) (*M*_w_ 350.43), phosphate buffered saline (PBS), acetic acid, sulforhodamine B (SRB), trypan blue, ribonuclease A (RNase) from bovine pancreas (*M*_w_ 13,700; solution of 50% glycerol), propidium iodide (*M*_w_ 668.39, purity ≥ 94%) and Triton X^TM^-100 were purchased from Sigma-Aldrich (St. Louis, MO, USA). Cholecalciferol (vitamin D_3_, *M*_w_ 384.65, purity ≥ 99%) was purchased from Alfa Aesar (Karlsruhe, Germany). Calcitriol (Rocaltrol, *M*w 416.64, purity ≥ 99%) was purchased from Selleck Chemicals (Munich, Germany). Uranyl acetate (dehydrate, 424.146 g/mol) was purchased from Electron Microscopy Sciences (Hatfield, UK). Dulbecco's Modified Eagle medium (DMEM) and Roswell Park Memorial Institute medium (RPMI) were acquired from Invitrogen Co. (Scotland, UK). Trichloroacetic acid (TCA) and Tris buffer were acquired from Merck (Darmstadt, Germany). SlowFade^®^ Gold Antifade Mountant with DAPI and LysoTracker^®^ Deep Red were purchased from Molecular Probes (Invitrogen Co., Scotland).

### Cell lines

Three different human cell lines were used in this work. The two human pancreatic cell lines, hTERT-HPNE (hTERT immortalized human pancreatic nestin-expressing normal duct-derived cells of the human pancreas) and S2-013 (well-differentiated tubular adenocarcinoma and moderately metastatic subline cloned from the human pancreatic tumor cell line SUIT-2), were provided by Prof. M. A. Hollingsworth (UNMC, Nebraska, USA) [40–41]. The human lung cancer cell line, A549 (nonsmall cell lung carcinoma), [11] was kindly provided by Dr. Gabriela Almeida (IPATIMUP). For cell culture purposes, the cell lines were maintained in DMEM (for pancreatic cells) or RPMI (for lung cells) medium, supplemented with 10% fetal bovine serum (FBS) at 37 °C in a humidified 5% CO_2_ incubator. When the cells reached 80% of confluence, they were trypsinized and subcultured.

### PLGA nanoparticle preparation

PLGA NPs were prepared using the single emulsion solvent evaporation technique. For that purpose, 10 mg of PLGA was dissolved in 0.1 mL of ethyl acetate, and for encapsulation, 1 mg of vitamin was added. PLGA NPs for the entrapment of coumarin-6 were also prepared by this method using 1% w/w of coumarin-6 (C6). 200 μL of an aqueous solution of 1% w/v Pluronic^®^F127 was added dropwise to the organic phase. Then, the solution was vortexed and emulsified by sonication at an ultrasonic frequency of 45 kHz. The emulsion was subsequently poured into 2.5 mL of 0.1% w/v Pluronic^®^F127 and stirred (800 rpm) at room temperature until complete evaporation of the organic solvent. The resulting suspension was filtered (0.2 μm, Millex-GP Filter Units, Merck Millipore, Germany) and incubated at 4 °C overnight to increase the NP stability. Then, the NPs were collected by centrifugation (14500 rpm, 30 min), and resuspended in ultrapure water. All formulations were prepared in triplicate.

Calcitriol-loaded PLGA NPs were freeze-dried to avoid the need to prepare particles whenever we conducted cell studies, and for the eventual future pharmaceutical applications. Lyophilization was carried out in a BenchTop^TM^ K series freeze-dryer (VirTis, NY, USA) at 5 × 10^−5^ bar and −95 °C for 48 h.

### PLGA nanoparticle physicochemical characterization

The size, polydispersity index (PDI), zeta potential, morphology, vitamin loading capacity and encapsulation efficiency were the parameters used to characterize the produced nanoparticles. The size distribution and zeta potential were determined by dynamic light scattering (DLS) and electrophoretic light scattering (ELS), respectively, using a ZetaSizer Nano ZS (Malvern Instruments, Worcestershire, UK). The size distribution was given by the PDI. DLS and ELS measurements were also performed to evaluate modifications in the PLGA NP size and zeta potential and possible particle aggregation over time under storage conditions (aqueous suspension stored at 4 °C) and after freeze-drying. The effect of sucrose (at a concentration of 1% w/v) used as a cryoprotective agent on the NPs stability during freeze-drying was also determined. The stability of the calcitriol in the NPs was evaluated by UV–vis spectrophotometry measurements after freeze-drying.

Unloaded and vitamin-loaded PLGA NPs were also analyzed for size and morphology by transmission electron microscopy (TEM) using a JEOL JEM 1400 microscope (Tokyo, Japan) at an accelerating voltage of 80 kV. The samples were deposited on copper grids (formvar/carbon on 400 mesh Cu from Agar Scientific) and negative-stained with 2% v/v uranyl acetate for 45 s. The grids were air-dried prior to TEM visualization [42].

Vitamin loading capacity (LC) and the encapsulation efficiency (EE) of PLGA NPs were further indirectly determined. For the quantification of the free vitamin, the NP suspension was centrifuged (14500 rpm, 30 min), and the supernatant analyzed. This step was conducted before organic solvent evaporation to ensure vitamin solubility. The sample was measured by UV–vis spectrophotometry at 265 nm, using a UV-1700 PharmaSpec UV–vis spectrophotometer from Shimadzu (Japan). The results were inferred from a calibration curve of known vitamin concentrations. All experiments were performed in triplicate.

### In vitro release studies

The in vitro release behavior of calcitriol entrapped in PLGA NPs was assessed over seven days. A sufficient amount of calcitriol-loaded PLGA NPs were resuspended in release buffer (PBS 0.01 M, pH 7.4) and divided into 7 aliquots. The aliquots were maintained at 37 °C and at determined set time points, and each aliquot was centrifuged at 14500 rpm for 30 min. Amicon^®^ Ultra-0.5 centrifugal filter devices (Merck Millipore, Germany) were used to remove PLGA degradation products and NPs. The release medium was freeze-dried and further reconstituted with ethanol 100% v/v for measurement by UV–vis spectrophotometry at 265 nm and the amount of released calcitriol was calculated from the calibration curve in ethanol. A solution of calcitriol in PBS was used as control to assess the stability of calcitriol in the release conditions over the seven days. All experiments were performed in triplicate.

### Cellular imaging studies

Laser scanning confocal microscopy (LSCM) was used to evaluate the NP in vitro uptake and morphological changes in S2-013, hTERT-HPNE and A549 cells. The cells were seeded in µ-chamber 12-well plates (ibidi, Germany) at a density of 1000 cells per well for 24 h prior to the experiment. This period, under normal conditions (5% CO_2_ humidified atmosphere at 37 °C), allows cells to adhere. The cells were then treated with 2.4 µM free calcitriol and entrapped calcitriol in C6-loaded PLGA NPs for 2 and 72 h. The lipophilic fluorescent dye C6 entrapped in the NP matrix does not leach during the experiment, allowing a fluorescent visualization of the uptake of PLGA NPs [30]. After the incubation period, the cells were rinsed with PBS and fixed using 4% paraformaldehyde for 15 min. The cells were then treated with LysoTracker^®^ Red (a marker of endo-lysosomal compartments) for 1 h. The cells were washed with PBS and mounted on a glycerol-based medium with DAPI for nuclear staining. Acquisitions were performed with a Leica TCS SP5 II confocal laser scanning microscope (Leica Microsystems, Germany) in emission mode. Untreated cells were also imaged as control. Different areas were analyzed and at least six images were acquired for each type of cell. The ImageJ JACoP “colocalization finder” plug-in was used for the determination of the Pearson coefficient (*r*), as a quantitative indicator of colocalization of the NPs in the lysosomes of cells.

### In vitro cytotoxicity studies

The effects of the calcitriol-loaded PLGA nanoparticles and free calcitriol on the cell growth of different human cell lines were evaluated by sulforhodamine B (SRB). This colorimetric method allows an indirect estimation of cell number by measuring cellular protein content [43].

The experiments were performed in 96-well assay plates, where exponentially growing cells were seeded for an incubation period of 24 h at a density of 1000 cells per well before treatment with free calcitriol, blank PLGA NPs and calcitriol-loaded PLGA NPs. The NP samples and free calcitriol were diluted in cell culture medium at eight final concentrations of calcitriol ranging from 0.005 to 3.2 µM, and the cells were incubated with these samples for 48 h and 72 h. A calcitriol stock solution of 3.2 mM was prepared in ethanol to ensure calcitriol solubility, but all samples of calcitriol alone contained at most 0.1% v/v ethanol. Due to the short half-life of calcitriol in cell culture medium, the supplemented medium was renewed daily [44]. After the 48–72 h incubation period, the cytotoxic effect was assayed by SRB, in a similar manner as previously described in [30]. The cells were fixed with 10% TCA for 1 h at 4 °C. The cell monolayers were then washed and stained with 50 µL SRB dye for 30 min. The cells were subsequently washed repeatedly with 1% acetic acid to remove any unbound dye. The cells were air-dried and the protein-bound stain was solubilized with 10 mM Tris solution. The SRB absorbance was measured at 560 nm using the PowerWave microplate reader (HT Microplate Spectrophotometer, BioTek). By comparing the measured absorbance of the wells containing the drug or the NPs with the measurements of the wells containing the untreated cells, it was possible to generate dose-response profiles and determine the concentration inhibiting the net cell growth by 50% (GI_50_). This step was perfomed following the incubation period, and subsequent comparison of these results with those obtained for cells that had been fixed at time zero (the time at which calcitriol/NPs were added).

Unloaded PLGA NPs and 0.1% of ethanol were added as a control to assess the effect on cell growth in control cells. Unexposed cells were also included in all assays as nontreatment controls (null controls). Two independent experiments were measured in triplicate.

### Cell cycle analysis

The cell cycle analysis was conducted by flow cytometry (FCM). The cells were seeded in T75 flasks at a density of 1 × 10^5^ cells/mL for 24 h prior to the experiment. The cells were then treated with 1.2 µM of free calcitriol and entrapped in PLGA NPs for 72 h. Due to the short half-life of calcitriol in cell culture medium, the supplemented medium with free calcitriol was renewed daily. Untreated cells were also used as a control. To reduce the effects of contact inhibition, control cells were adjusted to reach 60–70% confluence at the time of FCM analysis. After the incubation period, the cells were harvested and fixed with 70% v/v ethanol. The cells were then stained with a DNA staining solution (0.1% v/v TritonX-100, 20 µg/mL PI and 35 µg/mL of RNase A in PBS) at a cell density of 10^6^ cells/mL. FCM (FACSCalibur, BD Biosciences, CA, USA) was performed by plotting 12,000 gated events per sample. The data were subsequently analyzed by FlowJo 7.2 software (Tree Star, Ashland, USA). Three independent experiments were conducted.

### Statistical analysis

Statistical analysis was performed by using a two-tailed Student’s t-test, considering a 95% confidence interval. p-values lower than 0.05 were considered significant.

### Acronyms

**Table 4:** List of abbreviations used within the article.

Term	Abbreviation

drug delivery systems	DDS
dynamic light scattering	DLS
Dulbecco's Modified Eagle mMedium	DMEM
encapsulation efficiency	EE
electrophoretic light scattering	ELS
enhanced permeability and retention effect	EPR effect
fetal bovine serum	FBS
flow cytometry	FCM
Food and Drug Administration	FDA
half maximal growth inhibitory concentration	GI_50_
half maximal survival inhibitory concentration	IC_50_
loading capacity	LC
laser scanning confocal microscopy	LSCM
multidrug resistance	MDR
nanoparticles	NPs
phosphate buffered saline	PBS
polydispersity index	PDI
poly(glycolic acid)	PGA
propidium iodide	PI
poly(lactic acid)	PLA
poly(lactic-*co*-glycolic acid)	PLGA
Roswell Park Memorial Institute medium	RPMI
sulforhodamine B	SRB
trichloroacetic acid	TCA
transmission electron microscopy	TEM
ultraviolet–visible radiation	UV–vis
vitamin D receptor	VDR

## Supporting Information

File 1 Effects of calcitriol after 48 h and 72 h treatment on cell survival.
