# Supplementary material for: PLGA nanoparticles as a platform for vitamin D-based cancer therapy
Source: Beilstein J Nanotechnol. 2015 Jun 12;6:1306–18. doi: 10.3762/bjnano.6.135 (PMC4505177; doi:10.3762/bjnano.6.135)
Supplement: File 1 — Effects of calcitriol after 48 h and 72 h treatment on cell survival. [file Beilstein_J_Nanotechnol-06-1306-s001.pdf]

Supporting Information

for

PLGA nanoparticles as a platform for vitamin D-based cancer therapy

Maria J. Ramalho[1], Joana A. Loureiro[1], Bárbara Gomes[1], Manuela F. Frasco[1], Manuel A. N. Coelho[1] and M. Carmo Pereira[1]*

Address: [1]LEPABE, Department of Chemical Engineering, Faculty of Engineering, University of Porto, Rua Dr. Roberto Frias, 4200-465 Porto, Portugal

Email: Maria do Carmo da Silva Pereira - mcsp@fe.up.pt

* Corresponding author

Effects of calcitriol after 48 h and 72 h treatment on cell survival

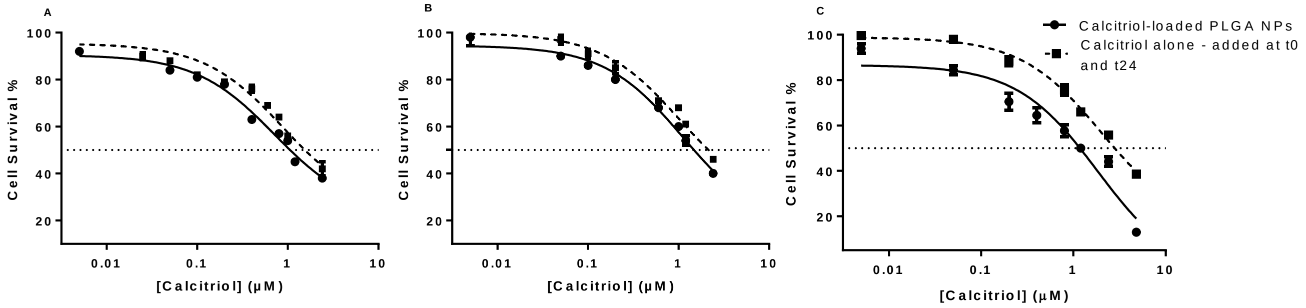

Figure S1: Cytotoxic effects of calcitriol free and entrapped in PLGA NPs after 48 h treatment on the cell survival of three human cell lines, (A) S2-013, (B) hTERT-HPNE and (C) A549, determined by SRB assay. Free calcitriol is represented with squares and dotted line; and calcitriol–NPs with spheres and solid line.

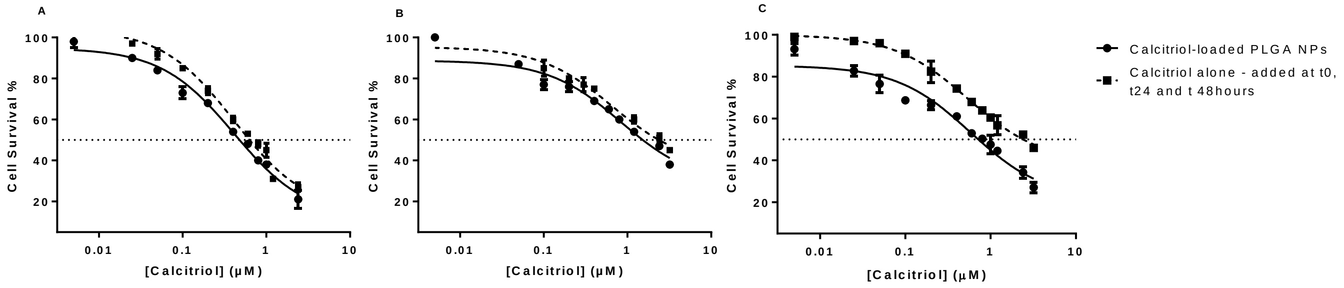

Figure S2: Cytotoxic effects of calcitriol free and entrapped in PLGA NPs after 72 h treatment on the cell survival of three human cell lines, (A) S2-013, (B) hTERT-HPNE and (C) A549, determined by SRB assay. Free calcitriol is represented with squares and dotted line; and calcitriol-NPs with spheres and solid line.
